# Enhancing Polymer Blend Compatibility with Linear and Complex Star Copolymer Architectures: A Monte Carlo Simulation Study with the Bond Fluctuation Model

**DOI:** 10.3390/polym16121626

**Published:** 2024-06-08

**Authors:** Juan J. Freire, Costas Vlahos

**Affiliations:** 1 Departamento de Ciencias y Técnicas Fisicoquímicas, Facultad de Ciencias, Avenida de Esparta s/n, 28232 Las Rozas-Madrid, Spain; 2 Chemistry Department, University of Ioannina, 45110 Ioannina, Greece; cvlahos@uoi.gr

**Keywords:** polymer blends, star polymers, Monte Carlo Simulations, compatibilization, bond fluctuation model, scattering function, random phase approximation

## Abstract

A Monte Carlo study of the compatibilization of A/B polymer blends has been performed using the bond fluctuation model. The considered compatibilizers are copolymer molecules composed of A and B blocks. Different types of copolymer structures have been included, namely, linear diblock and 4-block alternating copolymers, star block copolymers, miktoarm stars, and zipper stars. Zipper stars are composed of two arms of diblock copolymers arranged in alternate order (AB and BA) from the central unit, along with two homogeneous arms of A and B units. The compatibilization performance has been characterized by analyzing the equilibration of repulsion energy, the simulated scattering intensity obtained with opposite refractive indices for A and B, the profiles along a coordinate axis, the radial distribution functions, and the compatibilizer aggregation numbers. According to the results, linear alternate block copolymers, star block copolymers, and zipper stars exhibit significantly better compatibilization, with zipper stars showing slightly but consistently better performance.

## 1. Introduction

Polymer molecules typically do not blend well unless they possess specific groups facilitating their interaction [1,2]. However, the compatibility of a binary polymer blend can be significantly enhanced by introducing a third polymer, known as a compatibilizer, which serves as a mediator between different molecules [3,4,5].

The process of polymer compatibilization holds considerable practical importance in addressing various challenges associated with polymer materials. One notable application relates with plastics recycling, which has emerged as a thriving global market, generating cost-effective products for a wide array of uses in the packaging, construction, agriculture, and automotive industries [6]. Plastic waste encompasses both single-commodity plastics like polyethylene, terephthalate, or polyolefins, as well as mixtures of incompatible plastics [7]. Recycling single plastics is a straightforward endeavor, offering a cost-effective means to diminish waste footprints. Conversely, separating incompatible plastics incurs higher energy costs [8]. The mechanical recycling of the latter yields materials with large phase-separated domains, characterized by weak interactions between incompatible elements due to strong compositional gradients. This impedes effective polymer entanglements, resulting in suboptimal mechanical properties [9,10,11].

The incorporation of interfacial compatibilizers serves to mitigate these shortcomings by reducing the interfacial tension between incompatible units and facilitating the dispersion of one phase within another, thereby enhancing the mechanical performance of resultant polymer blends [12,13]. Often, compatibilizers take the form of diblock or multiblock copolymers, constituted by segments chemically akin to the constituent homopolymers or exhibiting affinity with them. These copolymers promote the miscibility of each polymer blend component at the interface. The architecture of the copolymer plays a pivotal role in determining its efficacy as a compatibilizer, influencing its alignment, arrangement, and conformation at the biphasic interface [13,14].

Linear diblock copolymers, for instance, can establish entanglements with both homopolymer domains, effectively bridging the interface [6,15]. However, they also tend to form micelles in the bulk of each one of the homopolymers, hindering diffusion at the interface [16,17]. Increasing the molecular weight of copolymer blocks decreases the critical micelle concentration (CMC), thereby diminishing compatibilization efficiency [18]. Alternatively, linear alternating block copolymers are positioned at the interface and function as molecular zippers, enhancing interface adhesion [6]. Triblock copolymers exhibit higher CMC values compared to diblock counterparts, with multiblock copolymers proving to also be efficient compatibilizers. Actually, the theory shows that when a copolymer chain crosses the interface more than once, we can expect a further reduction in the interface tension [15,16,19,20].

The design of next-generation compatibilizers hinges on a comprehensive understanding of the performance of various copolymer structures. Experimental and theoretical work [21], together with numerical simulations [22,23,24,25], are useful tools for reaching this goal. In this work, we utilize the bond fluctuation model [26] (BFM) to conduct Monte Carlo simulations, analyzing systems comprising a mixture of two homopolymers (A and B) alongside copolymers with diverse architectures and compositions. Our research focuses particularly on “zipper stars,” comprising two diblock copolymer arms joined in reverse order from the central unit or junction point, alongside two more homopolymer arms (A and B). This unique star architecture may yield higher CMC values compared to linear diblock or alternating copolymers, enabling the tethering of domains both across and along the interface without micelle formation.

**Figure 1:**
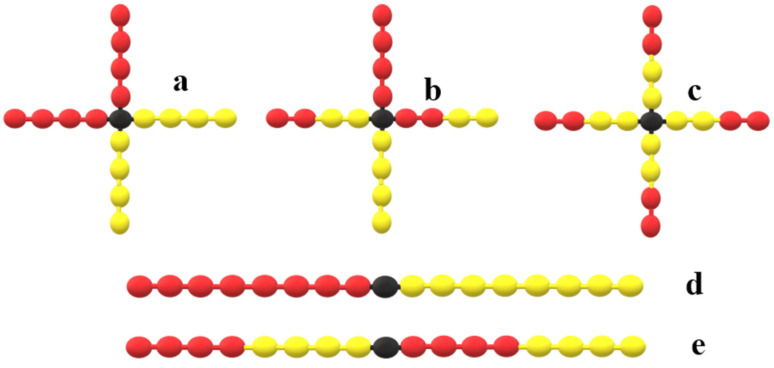
Schemes for the different copolymer architectures considered for compatibilization. (**a**) Miktoarm star, (**b**) zipper star, (**c**) star block copolymers, (**d**) linear deblock, (**e**) four-block alternating block copolymers.

To assess the compatibilization performance of zipper stars, we conduct Monte Carlo simulations of binary homopolymer mixtures (A and B) and ternary blends incorporating alternative copolymer architectures. In addition to zipper stars, we consider miktoarm stars (with two A arms and two B arms), stars with 4 AB diblock arms, linear diblock copolymers, and four-block alternating linear copolymers that can act as zipper molecules; see Figure 1.

While lattice models like the BFM offer computational efficiency and versatility, the direct calculation of forces or the interface tension is somehow challenging since these properties cannot be directly addressed. Nonetheless, we explore other various properties such as the repulsion energy decrease from a conformation equilibrated without considering the interactions and conformational averages of scattering functions, providing valuable insights into blend segregation. Additionally, we obtain unit type profiles along a longitudinal axis and radial distribution functions to elucidate the spatial distribution of the A and B polymer units within the blend. Moreover, we study the number of interconnections between compatibilizer molecules in order to understand how their topology influences the efficiency of the compatibilization mechanism.

## 2. Numerical Methods

Using the BFM, we investigate mixtures of *n* molecules, each composed of a given number of beads or sites, *N*, in a cubic lattice of length size, *L*, i.e., containing a total of *n_s_* = *L*^3^ sites. The length size is chosen to avoid most interactions between the replicas of the unit of the same molecule due to the customary consideration of periodic boundary conditions.

All the systems contain mixtures of homopolymers of types A or B. Ternary mixtures also include a minor number (about 20%) of compatibilizers, linear or star copolymers of different types, as described above, also with the same total number of units, *N*. All the molecules include a central unit that does not interact with the rest. The number of units in the blocks or arms within the copolymers is always symmetrically distributed. 

The length unit *b* represents the distance between adjacent lattice sites. Each unit site and the closest 26 positions satisfy the self-avoiding walk (SAW) condition for the BFM. Consequently, every unit can be considered to occupy eight sites, and the volume fraction can be calculated as *Φ* = 8*nN*/*L*^3^.

Bond lengths between units range between 2 and 10^1/2^, though the value 8^1/2^ is avoided since bonds of the type (±2 ±2 0) may cross each other. We include a distance-dependent [27] repulsion energy between A and B beads of non-bonded sites separated at a distance smaller than 10^1/2^, multiplied by a factor gauging the repulsion between units A and B, *β*, in units relative to the Boltzmann factor *k_B_T*. *β* is proportional to the bare Flory–Huggins parameter. According to the determination of the theta state in single-chain calculations with the BFM, *χ* ≅ 5*β* [28]. We take this relationship as a reference, though it is not totally clear that it can be applied to describe the behavior of other systems. Our repulsion interaction parameter does not try to mimic any specific polymer blend. 

The initial configurations are regular arrangements of extended molecules, leaving sufficient unoccupied beads between them to allow for an efficient and complete equilibration. The details about this procedure for systems including star polymer molecules are specified elsewhere [29]. Star copolymer molecules are included in adequately open regions selected at random but considering the desired volume fractions occupied by linear molecules, *Φ*_linear_, and stars, *Φ*_star_. The type of each particular homopolymer molecule is also selected at random, based on the required block architectures and the fixed goal for the fractions of molecules of each type. The simulations run over a fixed number of Monte Carlo elementary steps. They are simple bead jumps or displacements of a single unit to one of the lattice’s closest sites. A jump is accepted once the new bond distances and the SAW conditions are checked and the Metropolis criterion, which takes into account the change in energy between the old and new configurations, is satisfied. A Monte Carlo step is defined as *nN* elementary steps, after which there is an average single probability for each unit to change position.

Initial configurations are equilibrated using trajectories with no repulsion interactions, *β* = 0, over 1.8 × 10^6^ Monte Carlo steps. We have verified that the resulting systems are homogeneous and the individual properties of molecules are stable and consistent with the expected values. Subsequently, we set each one of the *β* values and extend the resulting trajectories over 1.8 × 10^7^ Monte Carlo steps, calculating the total repulsion energy, which is nearly proportional to the averaged number of repulsive contacts, and other properties of interest. These properties are collected once every 4000 steps. The curves of repulsion energies along the trajectories give useful information about how the A and B homopolymers tend to segregate in the presence of the different compatibilizers. The number of steps needed to reach a final equilibration is different for each one of the studied systems. The average properties correspond to the last 6 × 10^6^ steps in each trajectory, where the systems are equilibrated except when otherwise stated.

We calculate the conformational averages of individual properties for the different molecules such as the averaged squared radius of gyration, $\langle R_{g}^{2}\rangle$, the averaged quadratic distance between the central unit and end units, $\langle R_{ce}^{2}\rangle$, and the average of the asphericity, <*A*>, a property describing the molecular shape and defined as [30]

(1) $$A=\frac{\langle \sum_{i>j}^{3}\left(\lambda_{i}^{2}-\lambda_{j}^{2}\right)\rangle }{2\langle {\left(\sum_{i}^{3}\lambda_{i}\right)}^{2}\rangle }$$

where *λ_i_* is the *i*th eigenvalue of the radius of the gyration tensor.

Changes observed in these properties for the different systems along the trajectories give a clue of the process of equilibration for the different systems, while their averaged values in equilibrated samples can be compared with previously reported values.

We also calculate the total scattering intensity, obtained by the general expression

(2) $$I(q)=(1/n_{s})\sum_{i}^{n_{s}}\sum_{j}^{n_{s}}f_{i}f_{j}e^{iq\cdot R_{ij}}$$

where we can assign different values for the site contrast factors *f_i_*. With the choice of scattering factors 1, −1, and 0 for the sites occupied by the A and B units and the central and unoccupied sites, respectively [28], and taking into account that each unit in a site actually blocks eight sites in the lattice, we obtain the scattering function

(3) $$I_{AB}(q)=(8/n_{s})\left[\sum_{i_{A}}^{n_{s}}\sum_{j_{A}}^{n_{s}}e^{iq\cdot \left(R_{iA}-R_{jA}\right)}+\sum_{i_{B}}^{n_{s}}\sum_{j_{B}}^{n_{s}}e^{iq\cdot \left(R_{iB}-R_{jB}\right)}-\sum_{i_{A}}^{n_{s}}\sum_{j_{B}}^{n_{s}}e^{iq\cdot \left(R_{iA}-R_{iB}\right)}\right]$$

whose values can be compared with the results of the prediction of the random phase approximation [31,32] (RPA) for nearly homogeneous systems

(4) $$I_{AB}^{-1}(q)\approx 1/N\Phi_{A}P_{A}\left(q\right)+1/N\Phi_{B}P_{B}\left(q\right)-2\chi ,$$

 or

(5) $$I_{AB}^{-1}(q)=4/N\Phi P\left(q\right)-2\chi$$

for a symmetric blend of homopolymers composed of A or B units of similar volumes. *P*(*q*) is the form factor, or individual contribution to the scattering, of molecules in the absence of intermolecular interferences, *P*(*q*) = 1 for *q* = 0. This comparison allows us to explore how close the systems are to homogeneity at different distance ranges. Similarly, setting the scattering factors as 1 − *Φ*/8 for the sites occupied by A or B units and −*Φ*/8 for the rest of sites, we can detect any possible segregation, or inhomogeneity, in the global distribution of units with respect to the voids in the system, representing the solvent [28]. In systems where star polymers act as compatibilizers, setting factors as *Φ*_linear_ and −*Φ*_star_ for the sites occupied by linear molecules and stars, respectively, allows us to study the possible segregation between polymers of different morphologies. With the latter additional settings, we have verified that our equilibrated conformations are free from these two possible segregation effects.

We also compute the profiles of A and B units of both homopolymers and compatibilizers along the coordinate axis for which the higher maximum value for a given type is found in each system and the radial distribution functions, *g_AB_*(*r*), between a given homopolymer unit and those of a different type. Additionally, we build histograms of the number of interconnected compatibilizers in the different systems using the Python algorithm previously described in previous work [33,34]. We assume that two copolymer chains belong to the same network if two beads of a specific type are found within the distance range where bead interactions may occur.

## 3. Results and Discussion

The results correspond to systems with *N* = 49, *n* = 1275, and *L* = 100 unless otherwise indicated. For the reason that will be described below, we have performed some calculations with the alternative values *N* = 25, *n* = 352, and *L* = 52. Both sets of values are consistent with the volume fraction *Φ* ≅ 0.5 that is considered adequate to represent a BFM melt [35].

In Table 1, we show the results for the size and asphericity of individual molecules, *β* = 0. As expected, the ratios between the star and linear radius of gyration, as well as the asphericities in the melt state, are consistent with previous data [28,36] for single unperturbed molecules, and they do not show remarkable mixture effects.

We study the equilibration process after other values of positive *β* (net repulsion between A and B units) are introduced in a system that has been previously equilibrated with *β* = 0. Equilibration curves correspond to the values for the repulsion energy obtained along the trajectories for the different systems. For a given value of parameter *β*, these repulsion energies are approximately proportional to the number of repulsive interactions. We mainly consider symmetric mixtures with a similar number of molecules composed of A and B units, though we also have explored some asymmetric systems when the A and B homopolymers are in a ratio of about 30–70%.

The initial state corresponds to the final frame of equilibrated trajectories for the different homogenous systems. Therefore, initial energies are slightly different (less than 10%) in the pure homopolymer mixtures and in the mixtures including different types of compatibilizers. In any case, the energies are normalized according to these initial values. We compare the normalized values of the repulsion energy obtained for a system without a compatibilizer with the curves obtained for similar systems but with 20% of different types of copolymers.

**Figure 2:**
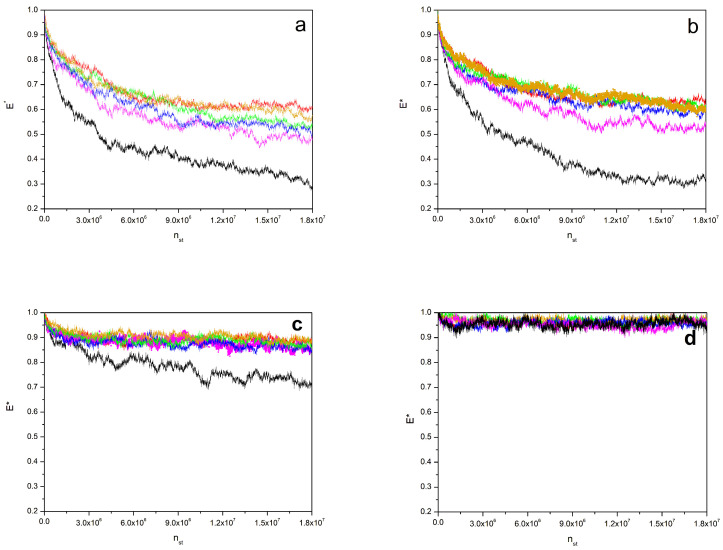
Equilibration of the normalized repulsion energy, E*, vs. the number of steps, *n_st_*, for systems of homopolymers with *N* = 49 and *Φ* ≅ 0.5 without and with ca. 20% of different compatibilizers. Last frame from bottom to up curves: black, binary mixture; magenta, linear block copolymers; blue, miktoarm stars; green, linear alternating copolymers; orange, star block copolymers; red, zipper stars composition. (**a**) *β* = 0.02, symmetric homopolymer composition; (**b**) *β* = 0.02, 30/70% composition of A/B homopolymers; (**c**) *β* = 0.01, symmetric homopolymer composition; (**d**) *β* = 0.005, symmetric homopolymer composition. Some lines are superimposed.

Figure 2 shows the data obtained with different values of the interaction parameter. Figure 2a corresponds to the results computed with *β* = 0.02. It is observed that, after 1.8 × 10^7^ simulation steps, the homopolymer binary mixture without a compatibilizer has not reached equilibration; the repulsion energy keeps decreasing, meaning that the different homopolymer molecules still tend to seek a further separation. According to the box length, *L* = 100, we expect that the repulsion energy may be able to decrease to about 0.1 of its value if the initial A/B interactions in three dimensions are constrained to occur in an ideal bidimensional interface. Moreover, we can verify that the systems containing 20% of the compatibilizer show a faster equilibration, with considerably higher final values of the repulsion energies and with an apparently equilibrated plateau, at least for the system containing the zipper stars. The highest final repulsion values correspond to the zipper star compatibilizers, followed by the star block copolymers, linear alternating copolymers, miktoarm stars, and diblock copolymers. We anticipate that a similar, or at least not significantly different, order is found for all the considered compositions and values of the repulsion parameter, though in some cases, the differences between systems are harder to distinguish and some curves are actually superimposed. A smaller decrease in the number of repulsive interactions implies a greater degree of mixing between the different homopolymers, suggesting that the described order corresponds to the performance quality of these different types of compatibilizers.

In Figure 2b, we show the results obtained with the same set of parameters for systems containing a ratio of 30/70 of the different homopolymers. In this case, most curves seem to reach their equilibration plateau in their final steps. The binary mixture without a compatibilizer shows higher final repulsion energy values than for the symmetric case, though, again, they are considerably smaller than those related with the systems with compatibilizers. The observed order of the compatibilizer performance does not differ much from the description provided above. The systems with star block copolymers, linear alternating copolymers, and miktoarm stars exhibit final results that are closer to those of the zipper stars, but the curve of the system with diblock copolymers is clearly lower. Therefore, an asymmetric composition tends to inhibit phase separation, and the compatibilization is somehow easier, showing a more similar performance for all the compatibilizer types except for the linear diblock copolymer.

In Figure 2c, we show similar curves for a symmetric mixture of homopolymers obtained with *β* = 0.01. Compared with the equivalent *β* = 0.02 system, the normalized final repulsion energies of the system without a compatibilizer are considerably higher, with a final reduction of less than 30% with respect to the initial value. However, the binary mixture is still far away from final equilibration. Therefore, segregation is clearly active at the end of the trajectory in this particular case. Nevertheless, all the systems with compatibilizers show a wide range of final nearly equilibrated values, with a practical superposition of the zipper and copolymer star curves and a final relative repulsion energy reduction of about 10–15%. The differences between the curves of the systems with 20% of compatibilizer molecules, are small, though they can still be noticed. Thus, we can observe that the curves corresponding to the systems with miktoarm stars and with diblock copolymers lie slightly below the rest, while the superimposed star block copolymer and zipper star curves lie slightly above the curve corresponding to the system with linear alternating copolymers, confirming the previously established performance order.

Figure 2d corresponds to the symmetric *β* = 0.005 system. We can observe that these systems exhibit a fast equilibration. In this case, the binary homopolymer mixture curve is superimposed with those of the systems with compatibilizers. The mean decrease in the relative repulsion energy during the equilibration process is smaller than 5%. Apparently, the value of the repulsion parameter is sufficiently small to avoid global segregation between different molecules.

**Figure 3:**
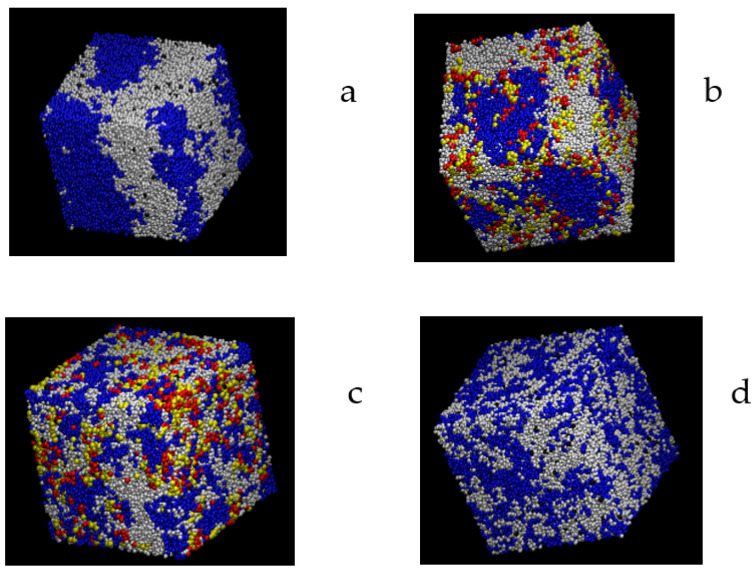
Snapshots of final frames for different systems: (**a**) homopolymer mixture, *β* = 0.02; (**b**) mixture with zipper stars, *β* = 0.02; (**c**) mixture with zipper stars, *β* = 0.01; (**d**) homopolymer mixtures, *β* = 0.005. Homopolymer units, blue and white. Star units: red and yellow.

The picture emerging from the repulsion energy equilibration plots is confirmed by the aspect of the snapshot of some equilibrated systems shown in Figure 3. Clear phase separation is observed for the *β* = 0.02 systems, though to a lesser degree in the presence of the compatibilizer molecules, located at the interfaces. The *β* = 0.01 system with the zipper stars is clearly more homogenous, which is also the case for the *β* = 0.005 system, even in the absence of compatibilizers. 

In order to have a more detailed description of the segregation between units of different types, we have obtained the scattering function for different systems with opposite contrast factors for A and B units, *I_AB_*(*q*). The RPA is used as reference. Low values of *q* correspond to global distances. According to the RPA approximation, a divergence in the scattering data for *q* = 0, characterizing phase separation, occurs when *χ* reaches the value $\chi_{PS}=2/N\Phi$. Taking into account the volume fraction and the molecular number of units considered in most calculations, this value would correspond to $\chi_{PS}$ = 0.0816. However, it should be noted that the considered volume fraction is actually close to representing the melt state in the BFM. Therefore, it seems adequate to define an effective volume fraction, $\Phi_{eff}=\Phi /\Phi_{melt}$, where *Φ_melt_* is the real volume fraction for which the melt state is reached. Therefore, we can assume that the phase separation occurs when $N\chi_{PS}=2/\Phi_{eff}$ or $N\chi_{PS}=2\Phi_{melt}/\Phi$.

We have performed fits of the low-range values of 1/*I_AB_*(*q*) vs. *q* to three- or four-order polynomials, choosing the results showing a smooth curve with the lowest correlation. The present results for the inverse scattering at low values of *q* are small in all cases, so extrapolations cannot be very precise. These extrapolations are included in Table 2 for the systems with only homopolymers and also for those with zipper stars and different values of *β.* They are compared with the results obtained for systems with *N* = 25 and similar values of the relevant parameter *χNΦ_eff_* or *βN*. The simulations with *N* = 25 were performed with the specific goal of studying this particular point, since they show greater values of the 1/*I_AB_*(*q*) and, therefore, their extrapolations to *q* = 0 are somehow more accurate. Negative extrapolated values imply a divergence of the scattering function and, therefore, the presence of phase separation.

In the case of the homopolymer systems without a compatibilizer, negative extrapolated values have been obtained for the systems corresponding to *β* = 0.05 and *β* = 0.02 and *N* = 25. This is also the case for the systems with a zipper star, though the results are closer to zero.

Consequently, the *N* = 25 data exhibit a closer approach to the limit of the phase separation for the systems with a compatibilizer, implying that these systems are more homogeneous. The *N* = 49 data are not conclusive due to the numerical difficulties inherent in the extrapolations of these smaller values.

The extrapolations of the inverse scattering functions to *q* = 0 for the systems with *N* = 25 are consistent with a phase separation parameter *β_PS_* between the values *βN* ≅ 0.25 and *βN* ≅ 0.5, i.e., *β* ≅ 0.005 and *β* ≅ 0.01 for *N* = 49, agreeing with the features observed in the repulsion energy equilibration curves. Moreover, according to this conclusion, $\Phi_{melt}=N\chi_{PS}\Phi /2≅1.25\beta_{PS}N$ is bracketed between the values *Φ_melt_* ≅ 0.31 and *Φ_melt_* = 0.62. This range includes the value *Φ_melt_* = 0.5 previously proposed for the BFM model [35] and the smaller value *Φ_melt_* = 0.36 resulting from the analysis of our previous BFM results for symmetric miktoarm polymers [28].

**Figure 4:**
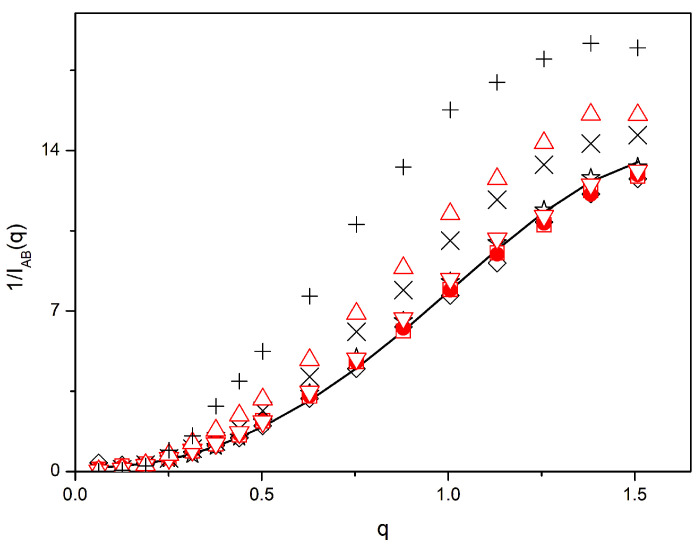
Inverse scattering for opposite contrast factors vs. the scattering variable. Black symbols: only homopolymers; rhombuses: *β* = 0; binary blends, +: *β* = 0.02; ×: *β* = 0.01; stars: *β* = 0.005. Red symbols: ternary system with 20% of zipper stars; circles: *β* = 0; triangles up: *β* = 0.02; triangles down: *β* = 0.01; squares: *β* = 0.005. Black line: RPA. Some symbols are superimposed with the RPA line.

In Figure 4, we show a detailed comparison of the inverse scattering results obtained with the *N* = 49 molecules with the RPA in the whole range of *q* values for the binary homopolymer mixtures and for systems containing 20% of zipper stars. The values obtained for *β* = 0, i.e., systems composed of homopolymers with or without homopolymer stars, are also included. The RPA curve for *χ* = 0 practically coincides with the results obtained for a linear homopolymer system, assuming it is a binary mixture of two non-interacting types of molecules with opposite contrast factors. The results obtained for the system of linear homopolymers and 20% of homopolymer stars with *β* = 0 are very close to the RPA curve and also to the results for the binary system, despise the presence of the star molecules with a smaller size.

Moreover, similar agreement with the RPA curve is found for the homopolymer mixture and with the system containing 20% of zipper stars when the segregation parameter is set as *β* = 0.005. However, we can observe differences with respect to the RPA curve for the case of the homopolymer mixture without a compatibilizer and *β* = 0.01, while the corresponding results obtained for the system with the zipper stars are also close to the RPA predictions. The results obtained for the mixtures with *β* = 0.02 are significantly different from those of the RPA curve for the systems with and without zipper stars, though agreement with RPA is clearly poorer when the compatibilizer molecules are absent. From these data, it can be concluded that the *β* = 0.005 systems with or without a compatibilizer show very small, if any, segregation between A and B units, while the *β* = 0.01 mixture with a compatibilizer also shows a significant degree of homogeneity. 

In the systems where we can observe deviations with respect to the RPA curve for higher values of *q*, the substitution of *χ* = 0 by a positive value reflecting incompatibility according to Equation (4) does not improve the agreement of the scattering curves with the RPA for intermediate or higher values of *q*. Actually, the theoretical RPA curves of the systems with interactions are displaced downwards as *χ* increases, i.e., in the direction opposed to the deviations observed at intermediate distances. It should be considered that systems with an important degree of segregation do not obey the RPA approximation. At intermediate values of *q*, or intermediate distances, scattering does not reflect the global segregation but rather the composition of smaller regions of the system enriched with A or B units. Therefore, the scattering of these regions tends to be smaller than for similar regions in non-segregated systems. This is the reason for the increase observed in our 1/*I_AB_*(*q*) results obtained for higher values of *β*. The comparison of these results with the prediction of the RPA actually serves as a clearer way to characterize the amount of phase separation, since only systems with a null or modest degree of phase separation are able to exhibit 1/*I_AB_*(*q*) values close to the RPA curve in the whole range of *q* values. According to Figure 4, the *β* = 0.005 systems and the *β* = 0.01 system with a compatibilizer comply with this criterion, while the binary homopolymer mixture with *β* = 0.01 and both *β* = 0.02 systems show a clear phase separation. This is consistent with our discussion for the equilibration curves. 

Systems with other types of compatibilizers show 1/*I_AB_*(*q*) values similar to those containing zipper stars since they are closer to the RPA curves than the corresponding mixtures without a compatibilizer, except for the case *β* = 0.005, where all systems, including the binary homopolymer blend, are close to the RPA. Therefore, at this level of comparison, we have not been able to find major differences for the scattering results of the different types of compatibilizers.

In order to perform a more precise quantitative analysis regarding this point, we have studied the small differences in the results obtained with the systems with the various types of compatibilizers with respect to the RPA. We assume that RPA predictions can be substituted by the results obtained for the equivalent systems with *β* = 0. As shown in Figure 4, these results are very close to the RPA prediction, and they incorporate the influence of lattice restrictions at low distances. Furthermore, they include the effects due to the inhomogeneity in topologies and sizes for the systems containing star compatibilizers. These effects can be relevant when dealing with small differences between similar values. Therefore, we calculate obtaining apparent results for the Flory–Huggins parameter that may show variations along the q range. According to our discussion on Figure 4, we expect that these differences are positive in a wide region of intermediate *q* values if the systems show segregation.

(6) $$\Delta I^{-1}\left(q\right)=I_{AB}^{-1}\left(q\right)-I_{AB}^{-1}\left(\beta =0\right)\left(q\right)=-2\chi_{app},$$

**Figure 5:**
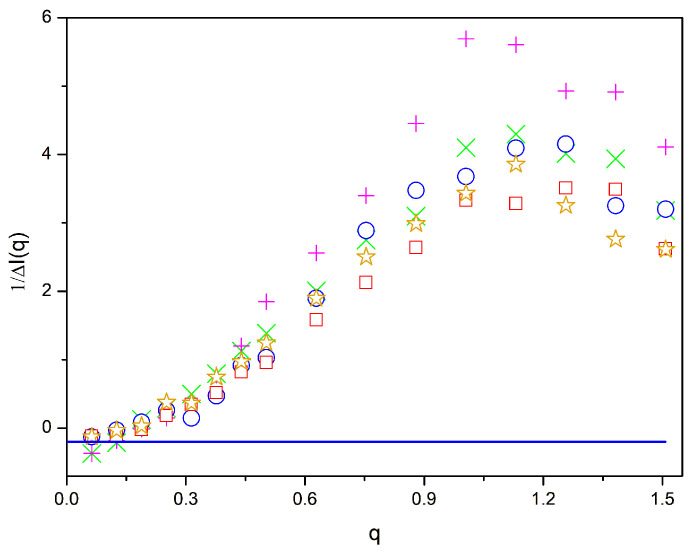
Values of 1/Δ*I*(*q*) vs. *q* for different systems with 20% of compatibilizers and *β* = 0.02. Magenta +: diblock copolymers; green ×: linear alternating copolymers; blue circles: miktoarm stars; orange stars: star block copolymers: red squares: zipper stars. Line: −2χ.

**Figure 6:**
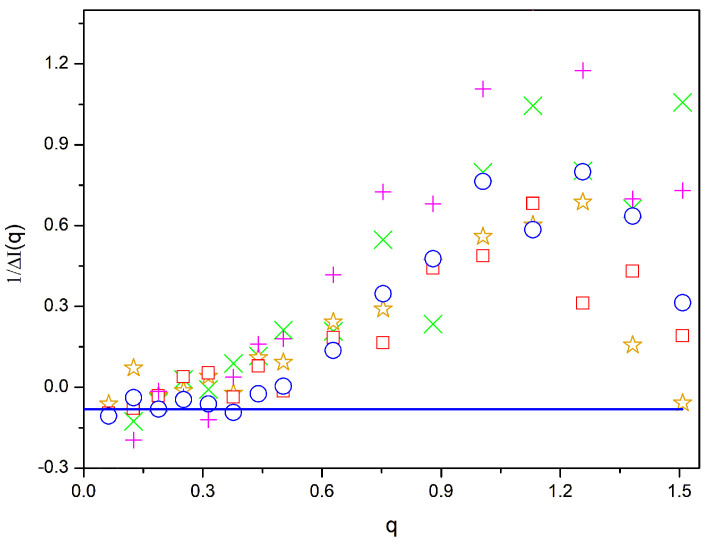
Values of 1/Δ*I*(*q*) vs. *q* for different systems with 20% of compatibilizers and *β* = 0.01. Magenta +: diblock copolymers; green x: linear alternating copolymers; blue circles: miktoarm stars; orange stars: star block copolymers: red squares: zipper stars. Line: −2χ.

**Figure 7:**
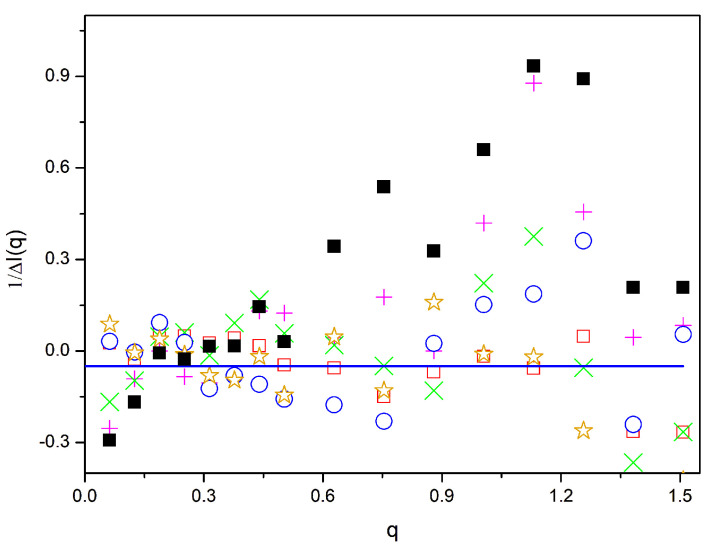
Values of 1/Δ*I*(*q*) vs. *q* for different systems, with 20% of compatibilizers and *β* = 0.005. Black squares: binary homopolymer blend; with a compatibilizer, magenta +: diblock copolymers; green ×: linear alternating copolymers; blue circles: miktoarm stars; orange stars: star block copolymers; open red squares: zipper star. Line: −2χ.

In Figure 5, Figure 6 and Figure 7, we present the values of $\Delta I^{-1}\left(q\right)$ for the *β* = 0.02, 0.01, and 0.005 systems, respectively. As shown in Figure 2a, Figure 3 and Figure 4, the *β* = 0.02 systems with a compatibilizer clearly exhibit phase separation, though to a lesser extent than for the binary blend. Differences with respect to the RPA curve are significant for all the systems with a compatibilizer in the range of intermediate values of *q*, reaching a maximum values at about six. At a higher *q*, differences become smaller, since some extra heterogeneity can be expected due to the junctions between blocks that have to be surrounded by A and B units at short distances. The differences in the intermediate q range are smaller for the systems with faster equilibration and higher final repulsion energies. In particular, the zipper stars show the best compatibilizer performance when considering this criterion.

The *β* = 0.01 systems with a compatibilizer show noticeably more homogeneous mixtures according to the results in Figure 2c and Figure 4. However, some small degree of global phase separation is still present, according to the extrapolations of the data for the system containing a zipper star to *q* = 0. In Figure 6, we observe more homogeneous mixtures than for *β* = 0.02. However, there is a clear departure from the RPA behavior. Thus, Figure 6 reveals a qualitative variation with a q value similar to that shown in Figure 5, though the observed difference values are significantly smaller with a $-2\chi_{app}$ maximum close to 1.2 in the intermediate range of q. This implies that the presence of regions with an excess of a given type of unit is still noticeable in the range of intermediate distances. The compatibilizer performance is noticeably poorer for the case of systems containing linear diblock copolymers, while it is slightly better for the zipper stars, confirming again the order described above for compatibilization.

Figure 7 shows the results for *β* = 0.005. In this case, Figure 2d and Figure 4 seem to indicate homogeneous mixtures in all the cases, including the binary homopolymer mixture. For this reason, we have also included the differences found for the system without a compatibilizer. In some systems, we again observe a variation in the differences along the *q* axis, although differences are always smaller than those found for higher values of *β*. The binary blend and the system containing linear diblock polymers show the greatest segregation effects, showing maximum values of $-2\chi_{app}$ of about 0.9. Only the star block copolymers and, particularly, the system with zipper stars show q-independent $\chi_{app}$ values that apparently fluctuate around zero with a small statistical fluctuation along the *q* range, confirming the homogeneity of these systems. Therefore, we conclude that these particular types of copolymers are able to guarantee homogeneous systems at all distance scales for systems close to phase separation. Although it was not explicitly discussed, a slight maximum in the differences in the inverse scattering data with respect to the RPA prediction could also be observed in the results obtained in our molecular dynamics simulations of atomistic models corresponding to real binary polymer mixtures with positive *χ* values close to 0 [37].

In Figure 5, Figure 6 and Figure 7, we have marked a line corresponding to $\Delta I^{-1}\left(q\right)=-2\chi ≅-10\beta$. According to what is described above, values below the line at *q* ≅ 0 correspond to a positive inverse scattering, marking global homogeneity. In spite of the statistical fluctuations in the differences shown above, most systems obtained with *β* = 0.005 exhibit values of −2$\chi_{app}$ clearly greater than −0.05 at low *q* values, i.e., they do not show global phase separation. However, most of the systems have compatibilizer values for *q* ≅ 0 above the −2*χ* line for *β* = 0.02, while they are very close to it for *β* = 0.01.

**Figure 8:**
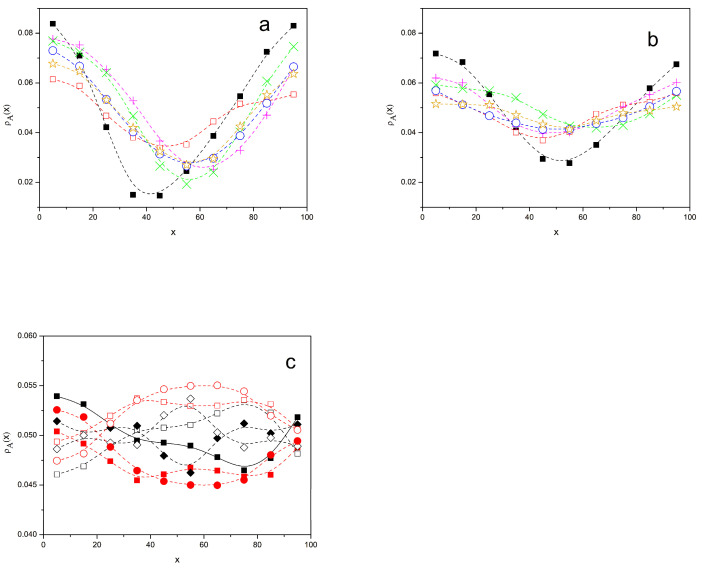
Profiles corresponding to the A monomers in different systems. (**a**) *β* = 0.02; (**b**) *β* = 0.01; (**c**) *β* = 0.005. (**a**,**b**) Black squares: binary homopolymer blend; systems with 20% compatibilizers, magenta +: diblock copolymers; green ×: linear alternating copolymers; blue circles: miktoarm stars; orange stars: star block copolymers; open red squares: zipper stars. (**c**) Solid symbols: A units; open symbols: B units. Black squares: binary homopolymer blend; Red squares: systems with 20% of zipper stars; Black rhombuses: linear chains with *β* = 0; Red circles: linear chains and 20% of stars with *β* = 0. Dash splines are guides for the eye, with *β* = 0.01.

**Figure 9:**
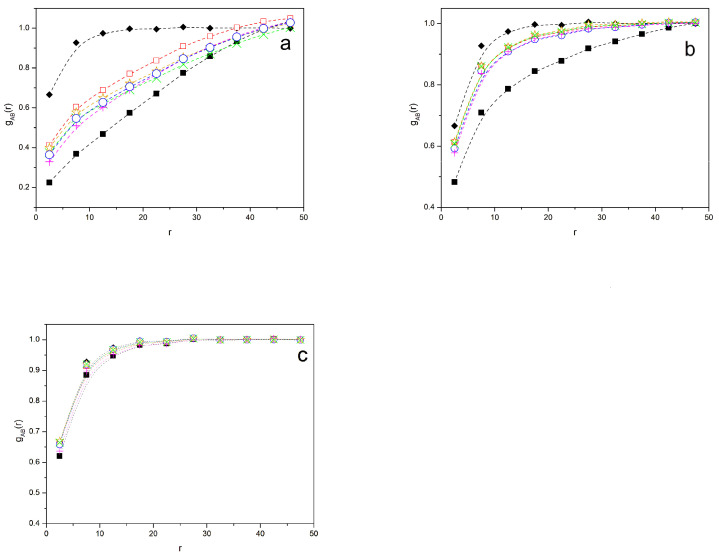
Radial distribution functions of B monomers with respect to an A unit for different systems. Black squares: binary homopolymer blend; systems with 20% of compatibilizers, magenta +: diblock copolymers; green ×: linear alternating copolymers; blue circles: miktoarm stars; orange stars: star block copolymers; open red squares: zipper stars. (**a**) *β* = 0.02; (**b**) *β* = 0.01; (**c**) *β* = 0.005. Black rhombuses: non-segregated homopolymer systems; *β* = 0 also included for comparison. Splines are guides for the eye.

**Figure 10:**
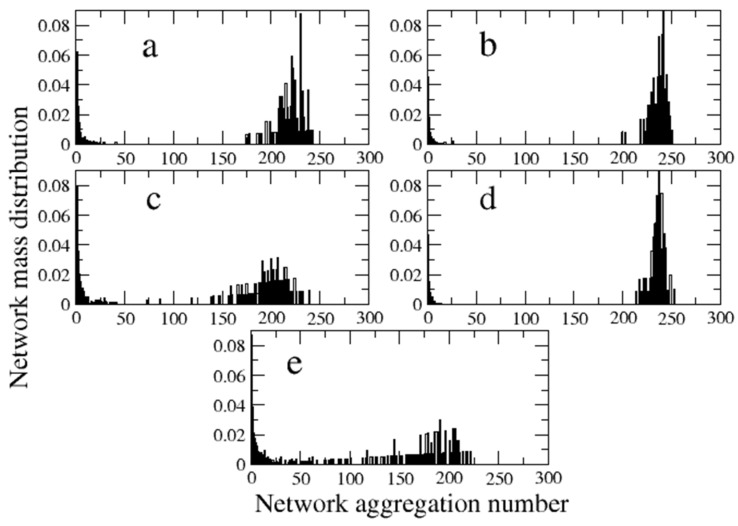
Distribution of the compatibilizers according to their aggregation number in interconnected networks for the systems with *β* = 0.02. (**a**) Zipper stars; (**b**) star block copolymers; (**c**) miktoarm stars; (**d**) linear alternating copolymers; (**e**) linear diblock copolymers.

Figure 8 shows the normalized profiles of the A units, along the *x* axis, *ρ*_A_(*x*), for the non-binary systems with *N* = 49 and *β* = 0.02, 0.01, and 0.005. The *x* axis is translated in each case so that the maximum density of units is shown at the smallest *x* value for all systems. For *β* = 0.02, in Figure 9a, it is verified that the system with zipper star compatibilizers exhibits a noticeably flatter profile and a higher minimum, i.e., in the region mainly occupied by the B units, which is consistent with the better compatibilization performance of these molecules for highly segregated systems. This confirms the tendency shown by repulsion energy equilibration curves and the scattering results. Similarly, we can also observe the same tendency for *β* = 0.01, as shown in Figure 9b. The system with zipper stars shows a flatter curve, though differences in the profiles obtained for the systems with compatibilizers are smaller. However, the profile of the binary system without a compatibilizer is considerably sharper. All these features are consistent with our previously discussed results for other properties. In Figure 9c, we show the profiles for the system without a compatibilizer and with zipper stars for *β* = 0.005 on a smaller scale. As expected for nearly or totally homogeneous systems, all the profiles are flat, fluctuating around the normalized mean value of 0.05 for all 20 of the considered values of *x*. In Figure 10, we also include the profiles for B units, which obviously show interchanged maximum and minimum values with respect to those of A. Moreover, we have included the profiles obtained for *β* = 0, i.e., in the absence of interactions, for the binary system and also for the system including stars in order to verify that the results for *β* = 0.005 are close to those of a totally homogeneous system. The differences between the results for all these different systems are small and can be attributed to statistical fluctuations.

Figure 9 shows the *g_AB_*(*r*) radial distribution functions for the different symmetric systems with N = 49 and *β* = 0.02, 0.01, and 0.005. The results obtained in the absence of interactions, *β* = 0, are included for comparison. It is noted that the system zipper star gives the closest results to the homogeneous system for *β* = 0.02, while the binary system without a compatibilizer shows a curve that is considerably below the plateau, even for moderately high values of r. For *β* = 0.01, all the systems with compatibilizers are closer to the homogeneous system, with small differences between them, while the system without a compatibilizer still lies well below the plateau for most *r* values. For *β* = 0.005, all values tend to agree with the homogeneous curve, though some marginal difference can still be noticed in the case of the binary blend. Again, all these features are consistent with the results observed for other properties.

The profile [21,22,23,24] and radial distribution function [25] results obtained using different simulation methods and theoretical models have also been previously reported, and they show features that are qualitatively consistent with those described here. We are not attempting a detailed comparison with them, since our work has been aimed at comparing different types of copolymers and, in particular, studying the performance of the zipper stars. 

**Figure 11:**
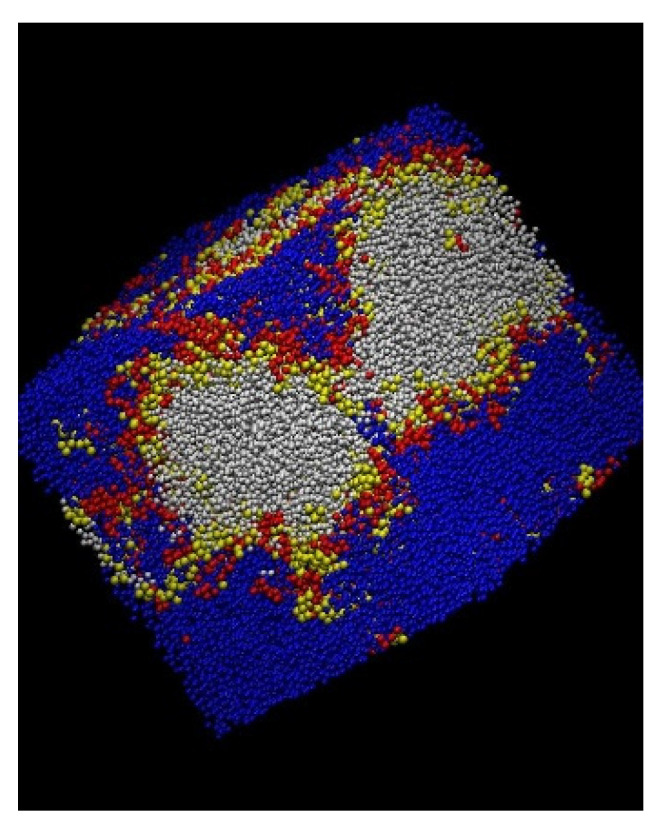
Snapshot of the final frame of the mixture with zipper stars for *β* = 0.05. Homopolymer units: blue and white. Star units: red and yellow.

In Figure 10, we show histograms of the percentage of the relative aggregation number, i.e., the number of compatibilizer molecules that are interconnected, forming a network for the choice of the repulsion parameter *β* = 0.02, for which phase separation clearly occurs with the presence of a well-defined interface. It can be verified that the molecules show a more efficient compatibilizing performance according to our results, namely, the zipper and copolymer stars and the linear alternating copolymers exhibit a peak near the total number of molecules. This result reveals a nearly total interconnection between compatibilizer molecules, as they tend to form a uniform layer along the interface. However, molecules with a weaker compatibilization capability manifest a greater number of isolated molecules and small clusters, which implies a smaller number of interconnected molecules at the interface. Therefore, a more efficient degree of compatibilization is associated with the possibility of establishing an extensive network of interconnected compatibilizer molecules along the interface, while their depletion from the interface in order to stay isolated, to aggregate, or to form micelles inside one of the phases is a crucial factor diminishing their effectiveness. The disposition of the zipper stars along the interface of a particularly segregated system, obtained with a higher choice of the interaction parameter, *β* = 0.05, is illustrated in Figure 11. The presence of the compatibilizer tends to restructure the flat interfaces. A more detailed analysis of this feature could be conducted in terms of a recently proposed method based on the determination of order parameters [38].

## 4. Summary and Conclusions

The present Monte Carlo simulations using the bond fluctuation model have allowed us to compare the degree of segregation present in binary mixtures of homopolymers and also in ternary systems including different types of copolymer compatibilizers as a function of the value of a parameter introduced to describe the repulsion between units of different types, proportional to the Flory–Huggins parameter. We have considered linear and star block copolymers, miktoarm stars, and linear and star copolymers with alternating blocks in the different parts of the molecules, designed to provide a zipper effect. We have studied equilibration curves based on the repulsion energy values calculated on the basis of trajectories starting from the conformation previously equilibrated without interactions. We have also computed the scattering functions obtained with opposite types of contrast factors for units of different types, the profiles of different types of units along an axis, and the radial distribution functions of a type of unit with respect to a unit of a different type.

Discussing the results obtained from these properties, we have concluded that there are noticeable differences in the performance of the different compatibilizers. The degree of segregation in each system is apparent from the aspect of the repulsion energy equilibration curves. Similarly, we observe segregation in systems where a global phase separation is expected according to the random phase approximation, with a divergence of the scattering function for small values of the scattering variable. When a relatively high repulsion energy between units is introduced, segregation is found in the binary and ternary systems, though it is present in a clearly lesser degree in the latter systems. Moreover, we find a consistent order of performance of the compatibilizers, especially for the highly segregated systems. Zipper stars are able to induce some homogeneity in these systems in a more efficient way, followed by star block copolymers and linear alternating copolymers. These structures can form an extensive network of interconnected molecules along the interface. Miktoarm stars and, even more distinctly, diblock copolymers are shown to be less efficient. Some of these latter molecules can stay out of the interface, either isolated or forming small aggregation clusters or micelles within one of the phases.

When the interaction between units is reduced to a smaller value, we find that global segregation suffers a drastic reduction in all the systems with compatibilizers, though, according to the scattering results, it persists in the intermediate range of distances. However, the binary systems show phase separation in the whole range of distances. Systems are predominantly homogeneous in the cases where the scattering function does not diverge, though some marginal segregation is still apparent at intermediate distances in some of them, particularly in the binary mixtures and ternary mixtures with diblock copolymers. Nevertheless, we do not find any evidence of segregation at any distance scale in the system with zipper stars.

## Figures and Tables

**Table 1:** Values of the averaged quadratic radius of gyration, *R_g_*, quadratic distances between the center and ends, *R_ce_*, and asphericities, *A*, in systems of linear chains and mixtures with 20% of stars. *β* = 0, *N* = 49, *n* = 1275, and *L* = 100.

	<*R_g_*^2^> (Linear)	<*R_g_*^2^> (Star)	<*R_ce_*^2^> (Linear)	<*R_ce_*^2^> (Star)	<*A*> (Linear)	<*A*> (Star)
linear	77.4	-	226.5	-	0.526	-
mixture	77.1	51.8	225.7	114.2	0.526	0.248

**Table 2:** 1/*I_AB_*(*q*) extrapolated to *q* = 0, for binary mixtures of linear homopolymers and for ternary mixtures also containing ca. 20% of zipper stars and different *N* values. *χ_PS_*, is indicated.

	*β* = 0.05	*β* = 0.02	*β* = 0.01	*β* = 0.005
*N* = 25, *χ_PS_* = 0.16				
binary mixtures	−1.2 ± 2.2	−1.3 ± 0.4	0.19 ± 0.23	-
ternary mixtures	−0.5 ± 0.3	−0.8 ± 0.1	0.55 ± 0.35	-
*N* = 49, *χ_PS_* = 0.08				
binary mixtures	_	0.11 ± 0.79	0.04 ± 0.07	0.05 ± 0.11
ternary mixtures	_	0.03 ± 0.08	−0.06 ± 0.2	0.08 ± 0.05

## Data Availability

The original contributions presented in the study are included in the article, further inquiries can be directed to the corresponding author.
